# Deep clustering using 3D attention convolutional autoencoder for hyperspectral image analysis

**DOI:** 10.1038/s41598-024-54547-2

**Published:** 2024-02-20

**Authors:** Ziyou Zheng, Shuzhen Zhang, Hailong Song, Qi Yan

**Affiliations:** 1 College of Communication and Electronic Engineering, Jishou University, People’s South Road, Jishou, 416000 Hunan China; 2 Key Laboratory of Visual Perception and Artificial Intelligence, Hunan University, Lushan Road, Changsha, 410000 Hunan China

**Keywords:** Computer science, Information technology, Computer science, Information technology

## Abstract

Deep clustering has been widely applicated in various fields, including natural image and language processing. However, when it is applied to hyperspectral image (HSI) processing, it encounters challenges due to high dimensionality of HSI and complex spatial-spectral characteristics. This study introduces a kind of deep clustering model specifically tailed for HSI analysis. To address the high dimensionality issue, redundant dimension of HSI is firstly eliminated by combining principal component analysis (PCA) with *t-*distributed stochastic neighbor embedding (t-SNE). The reduced dataset is then input into a three-dimensional attention convolutional autoencoder (3D-ACAE) to extract essential spatial-spectral features. The 3D-ACAE uses spatial-spectral attention mechanism to enhance captured features. Finally, these enhanced features pass through an embedding layer to create a compact data-representation, and the compact data-representation is divided into distinct clusters by clustering layer. Experimental results on three publicly available datasets validate the superiority of the proposed model for HSI analysis.

## Introduction

Hyperspectral image (HSI) consists of numerous narrow and contiguous spectral bands that can capture subtle spectral variations between ground objects, which is beneficial for several remote sensing applications^1–7^. Most of these applications require classifying every pixel in the scene^8,9^. However, labeling HSI requires a lot of resources and effort, which may impose difficulties to fulfill our targets including object detection and classification. Therefore, developing an HSI method with less reliance on labeled samples is crucial.

The downscaling of HSI is necessary due to its property of rich spectral features. The mainstream dimensionality reduction methods include two kinds: feature extraction and band selection. Dimensionality reduction based feature extraction is to map the data into a low-dimensional space by projection transformation, and use the features in the low-dimensional space to participate in the subsequent processing. Classical methods include principal component analysis^10^, linear discriminant analysis^11^, etc.. Band selection is to select a subset of features directly from the original feature set to replace all the features and some of the more famous methods, such as band selection based on maximum information entropy^12^, and band selection method based on distance metric^13^.

The use of classifiers to accomplish HSI classification using only the spectral information of the data has been widely explored in early related studies, and many pattern recognition techniques, including Bayesian discrimination^14^, support vector machines^15,16^, decision trees^17^, and sparse representations^18^, have been proven to be effective HSI classifiers in a wide range of studies. However, since these methods only consider spectral features, the classification results conducted by them commonly contain a lot of noise.

To overcome this problem, many spatial-spectral feature extraction for HSI classification have been proposed. For example, Zhang et al.^19^ proposed an active learning adaptive multi-view technique to achieve HSI classification by incorporating spectral information and spatial information from segmentation maps into each view. Hong et al.^20^ proposed an iterative multi-task regression method to learn a low-dimensional subspace by considering both labeled and unlabeled samples.

Deep learning methods have received a lot of attention in recent years. Among deep learning methods, convolutional neural networks (CNNs) are widely used and typically comprise millions of trainable parameters. When optimized effectively, CNNs improve the quality of extracted features and enhance classification performance. These deep learning approaches are adopted in various applications, such as image classification, object detection,etc. Sharma et al.^21^ and Hamida et al.^22^ introduced the pioneering 2D-CNN and 3D-CNN approaches tailored for HSI classification. Dong et al.^23^ performed a weighted fusion of features with CNN and graph attention networks. These methods use deep learning to extract features that are more comprehensive than manual features and consistently outperform traditional methods in classification performance. However, due to the limited samples of HSI, it is difficult for supervised deep neural network (DNN) to take full advantage of HSI processing.

The challenge of scarce labeled HSI data and the associated performance issues with deep neural networks (DNNs) have been mitigated through the application of unsupervised DNN techniques. Chen and colleagues^24^ utilized a deep autoencoder (DAE) for classifying HSI, incorporating principal component analysis (PCA) for dimension reduction along with spatial-spectral information. Likewise, Mei and co-authors^25^ introduced a 3D convolutional autoencoder (3D-CAE) designed to extract unsupervised spatial-spectral features. The proposed methodology involves the integration of 3D operations, including convolution, pooling, and batch normalization, thereby preserving the intrinsic 3D structure of HSI. However, due to the inherent characteristics of unsupervised DNN models, they generally lack support for end-to-end training.

Unsupervised clustering algorithms are frequently employed to categorize data into clusters according to sample similarities. Clustering is the process of partitioning a dataset into different classes or clusters according to a specific criterion, so that the similarity of data objects within the same cluster is as large as possible, while the differences of data objects not in the same cluster are as large as possible. That is, after clustering, the data of the same class are gathered together as much as possible, and the data of different classes are separated as much as possible. The K-means algorithm^26^ organizes samples by iteratively computing the distance between each sample and the cluster center. Nonetheless, K-means is susceptible to uneven clustering outcomes owing to its initial random selection of sample points. Consequently, an enhanced version known as Xmeans^27^ has been developed to tackle this issue. Moreover, various clustering algorithms, including spectral clustering^28^ and subspace clustering^29,30^, have undergone extensive research.

Studies have shown that traditional clustering algorithms combined with DNN, called deep clustering^31^, improve the effectiveness and accuracy of clustering algorithms by jointly optimizing DNN parameters and clustering results. The deep clustering method involves employing a DNN as a feature extractor and incorporating a layer designed to induce a clustering effect within the model structure. This specific layer is utilized to obtain clustering results, subsequently guiding the parameter adjustments of the DNN based on these clustering results. Deep clustering enables DNNs to learn more representative and discriminative features for clustering. In addition, feature representation and clustering accuracy^32^ is significantly enhanced through iterative updates of the DNN and clustering results. Deep clustering is categorized based on the DNN structure into autoencoder-based and separated network-based deep clustering^33^. Autoencoder-based deep clustering methods usually induce a clustering layer between the encoder and the decoder, and directly use the clustering results to guide the network parameters, while deep clustering based on separated networks separates the DNN from the clustering, and the DNN is trained by using the pseudo-labels obtained from the clustering. DeepCluster^34^ exemplifies a representative separated network-based deep clustering method that alternates between K-means and network loss functions for model optimization. In contrast, a deep clustering network (DCN)^35^, an instance of an autoencoder-based deep clustering network, embeds clustering methods into the autoencoder for collaborative training purposes.

Utilizing the deep clustering technique in HSI has been studied by some researchers. Nalepa et al. applied deep clustering technique in HSI segmentation for the first time by 3D convolutional autoencoder(3D CAE)^36^. Meanwhile, Tulczyjew et al. extended their experiments. They went deeper by proposing an asymmetric recurrent neural network (RNN)-based AE^37^ for deep clustering, utilizing recurrent neural networks with the replacement of convolutional operations. Experimental results indicated that both models were able to achieve good performance.

However, all existing deep clustering models have huge room for improvement in clustering accuracy. The attention mechanism, as a deep learning structure, is able to automatically learn and calculate the magnitude of the contribution of the input data to the output data. There are many studies that have added attention mechanisms to deep learning models to further enhance the modeling performance. Mei et al.^38^ Integrating RNN and CNN with attention mechanisms to capture both spectral and spatial correlations. Ribalta et al.^39^ utilized a CNN with an attention mechanism for band selection, which was able to improve the feature extraction accuracy of the CNN without affecting the training speed and classification ability. However, in the study of HSI, the current mainstream attention mechanisms have a two-branch structure, which is unable to jointly process spatial-spectral features.

In light of the comprehensive analysis presented above and with the aim of reducing the model’s dependence on sample quantity and achieving a more rational extraction of spatial-spectral information from HSI, this paper introduces a novel deep clustering framework incorporating an attention mechanism. First, HSI undergoes dimensionality reduction (DR) using the PCA and t-SNE method to eliminate redundant spectral bands. Subsequently, the reduced-dimensional HSI is fed into the 3D attention convolutional autoencoder (3D-ACAE) for feature extraction. The 3D-ACAE comprises an encoder and a decoder, incorporating 3D convolutional layers and 3D spatial dropout to prevent overfitting. An attention module is introduced in the encoder after the second convolutional layer to refine the extracted features. The features extracted by the encoder are flattened into one-dimensional vectors and compressed using a fully connected layer called the embedding layer. Finally, the output of the embedding layer is fed into the model’s clustering layer to obtain the final clustering results. The clustering layer is optimized using the backpropagation algorithm, initialized with the K-means algorithm, and utilizes the Kullback-Leibler (KL) divergence to calculate the clustering loss. Clustering labels are rearranged using the Hungarian algorithm to obtain accurate evaluation metrics.

The main contributions of this paper are as follows.

1. We propose the integration of a 3D-ACAE into a deep clustering framework. The 3D-ACAE incorporates a spatial-spectral attention module, playing a vital role in the encoding process. This integration results in improved precision and accuracy in feature extraction.
2. We implement a approach that combines PCA and t-SNE for preprocessing HSI data. By combining linear and non-linear methods for preprocessing, the DNN can learn more accurate features, improving its overall preformance.
3. The experimental results indicate that the application of deep clustering methods in HSI analysis has led to improvements, yielding clustering results superior to both traditional clustering methods and newly proposed deep clustering methods.

The rest of this article is organized as follows. “Related works” section provide an overview of related work, “Proposed methods” section presents a detailed description of our proposed method. “Experimental results and analysis” section illustrates experimental results to validate the proposed method, and “Conclusion” section concludes the findings and discusses future perspectives.

## Related works

### Deep clustering

Deep clustering is a novel approach that combines DNNs with conventional clustering techniques, and it has been extensively studied in computer vision and other domains. The primary characteristic of deep clustering is its ability to optimize DNN parameters by employing loss functions from clustering. This study focuses on using the autoencoder-based deep clustering structure.

The deep clustering network (DCN), an AE-based deep clustering^35^, utilizes a deep autoencoder network to reduce dimensionality and acquire K-means-compatible features. It optimizes the clustering and dimensionality reduction tasks concurrently within a unified framework, as expressed in (1):

1 $$\begin{aligned} \begin{aligned} \min _{\textbf{I},\left\{ \textbf{s}_{i}\right\} } \sum _{i=1}^{{\varvec{M} \varvec{N}}}\left( \ell \left( g\left( f\left( \textbf{A}_{i}\right) \right) , \textbf{A}_{i}\right) +\frac{\gamma }{2}\left\| f\left( \textbf{A}_{i}\right) -\textbf{I s}_{i}\right\| _{2}^{2}\right) \\ \text{ s. } \text{ t. } \textbf{s}_{j, i} \in \{0,1\}, \textbf{1}^{T} \textbf{s}_{i}=1, \forall i, j, \end{aligned} \end{aligned}$$

where $f(\cdot )$ and $g(\cdot )$ denote non-linear mapping functions for the encoder and decoder respectively. $l(\cdot )$ represents the reconstruction loss function define as $l(\textbf{A}_{i},\textbf{B}_{i})=\left\| \textbf{A}_{i} -\textbf{B}_{i} \right\| _{2}^{2}$, where $\mathbf {A_{i}}$ is the original sample, $\textbf{B}_{i}$ is the reconstructed sample. **I** denotes the centroid matrix, with its *i*-th column representing the *i*-th cluster centroid. $s_{i}$ is the assignment vector with only one nonzero element. The objective function’s first and second itemss are the network model loss and the clustering assignment loss, respectively, with $\gamma$ being the trade off parameter.

### 3D convolutional autoencoder

Autoencoder (AE) is an unsupervised neural network model that learns implicit features from input data and reconstructs the original input data using these learned features. AE primarily consists of an encoder and a decoder. The encoder compresses the input into a compressed feature representation, and the decoder reconstructs these into output data closely resembling the initial input data.

The convolutional autoencoder (CAE) is a variant of AE that employs convolution layers instead of fully connected ones in its framework. This feature makes CAE more suitable for processing 2D and 3D data with spatial patterns, as it does not force the input data into a 1D vector.

The 3D-CAE is implemented with an encoder and a decoder. The encoder uses standard convolution operations instead of fully-connected layers. Given an input $\varvec{I} \in \mathbb {R}^{L \times H \times W}$, using kernel $K \in \mathbb {R}^{l \times h \times w}$ ($l\le L$, $h \le H$ and $w\le W$) for 3D convolution, the output can be defined as follows:

2 $$\begin{aligned} \begin{aligned} O^{x,y,z} = b + \sum _{p=0}^{l-1} \sum _{q=0}^{h-1} \sum _{r=0}^{w-1} K^{p,q,r}I^{x ^\prime +p, y ^\prime +q,z^\prime +r}\\ \text {where } x^\prime = x \cdot s_{x};^\prime = y \cdot s_{y};z^\prime =z\cdot s_{z} \end{aligned} \end{aligned}$$

where $O^{x,y,z}$ denote the (*x*, *y*, *z*)-th element of output $O \in \mathbb {R}^{L^{\prime }\times H^{\prime }\times W^{\prime }}$, $x \in [1, L^{\prime }]$, $y \in [1, H^{\prime }]$, and $z \in [1, W^{\prime }]$. *b* denotes the bias of the 3D convolution layer, $(s_{x}, s_{y}, s_{z})$ are the step sizes of the convolution kernel *K* in the three dimensions. $L^{\prime }$, $H^{\prime }$ and $W^{\prime }$ represent the sizes of output *O* and are defined as:

3 $$\begin{aligned} \begin{array}{lcr} L^{\prime }=\left\lfloor \frac{L-l}{s_{x}}\right\rfloor +1 ,\qquad&\qquad H^{\prime }=\left\lfloor \frac{H-h}{s_{y}}\right\rfloor +1 , \qquad&\qquad W^{\prime }=\left\lfloor \frac{W-w}{s_{z}}\right\rfloor +1 \end{array} \end{aligned}$$

where $\left\lfloor \cdot \right\rfloor$ means the round-to-zero process.

The decoder component employs a 3D transposed convolution to reconstruct the image, which is the inverse of convolution. Transposed convolution maps the input from a low-dimensional space to a high-dimensional space. This is achieved by zero-padding the input to create an intermediate input larger than the desired output size. The intermediate input is then convolutionally filtered to obtain the final output.

### PCA and t-SNE

PCA technique is widely used for reducing data dimensionality in various research domains. PCA linearly transforms the original linearly correlated high-dimensional data into linearly uncorrelated low-dimensional data. This process identifies the projection direction by maximizing the projected data variance for the original data set. Consequently, the newly projected data retains the most relevant information from the original data while discarding redundant components. The general formula for PCA can be summarized as follows:

4 $$\begin{aligned} {\textbf{C}}= & {} \frac{1}{n} {\textbf{X}_{c}^{T} \textbf{X}_{c}} \end{aligned}$$

5 $$\begin{aligned} \textbf{Y}= & {} \textbf{X}_{c} \textbf{W}\end{aligned}$$

where $\textbf{C}$ is the covariance matrix, $\textbf{X}_{c}$ is centered data matrix, $\textbf{W}$ is projection matrix, which with each column being one of the selected eigenvectors of $\textbf{C}$, $\textbf{Y}$ is reduced-dimensional data matrix and *n* is the number of samples.

Notably, t-SNE is a powerful dimensionality reduction technique for visualizing high-dimensional datasets by projecting them into a lower-dimensional space, typically two or three dimensions. t-SNE preserves the pairwise distance relationships between individual samples in the reduced feature space by constructing probability distributions for the high-dimensional samples. In these distributions, similar samples (represented by high probabilities for nearby points) are more likely to be chosen, while different samples (with low probabilities for distant points) are less likely to be selected. It achieves this by computing the conditional probabilities $p_{j|i}$ that measure the similarity between data point *i* and data point *j* using a Gaussian kernel:

6 $$\begin{aligned} p_{j|i}= exp\left( \frac{-\left\| x_i - x_j \right\| ^{2}}{2 \times \sigma _{i}^{2}}\right) \end{aligned}$$

where $x_i$ and $x_j$ are the data points, and $\sigma _{i}$ is a bandwidth parameter chosen for each data point to adjust the scale of the Gaussian distribution. Next, t-SNE defines a similar distribution for the points in the low-dimensional embedding, computing the conditional probabilities $q_{j|i}$ for the lower-dimensional space:

7 $$\begin{aligned} q_{j|i} = \frac{(1 + ||y_i - y_j||^2)^{-1} }{Z_i} \end{aligned}$$

where $y_i$ and $y_j$ are the mapped points in the lower-dimensional space, and $Z_i$ is a normalization constant. Then, define the similarity between data points in the low-dimensional space as a joint probability:

8 $$\begin{aligned} q_{ij} = \frac{q_{j|i} + q_{i|j}}{2n} \end{aligned}$$

where n is the number of samples. Finally the KL divergence between the conditional probabilities in the high and low dimensional spaces is calculated to measure the difference between them. The KL divergence is then minimised by gradient descent to find the best low-dimensional representation.

## Proposed methods

**Figure 1:**
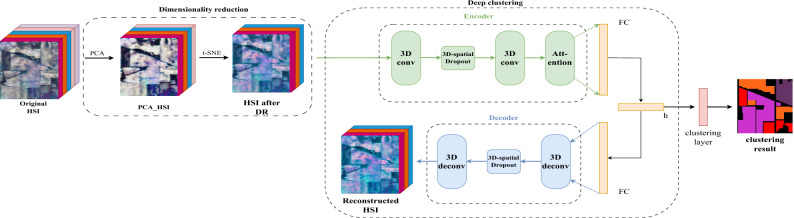
Structure of the 3D-ACAE. Where h is the embedding layer, FC is the fully connected layer, DR means dimensionality reduction.

The structure of the proposed method is shown in Fig. 1, which consists of two step: (1) HSI dimensionality reduction by combinating PCA and t-SNE and (2) the integration of 3D-ACAE in deep clustering. The following subsections describe the two main steps in detail.

### Dimensionality reduction by PCA and t-SNE

Given an original HSI $Z \in \mathbb {R}^{L\times H\times W}$, the reduced HSI $Z^{\prime } \in \mathbb {R}^{L\times H\times W^{\prime } }$ is obtained by using the combined PCA and t-SNE method, where *L* and *H* represent the sizes of two spatial dimensions, *W* denotes the original size of spectral dimension and $W^{\prime }$ means the size of the spectral dimension after reduction. After linear dimensionality reduction and manifold learning, the original HSI retains the utmost critical information within it. The PCA reduced the original HSI, and t-SNE reduced it further. In this study, we set $W^{\prime }$ to 60.

### Integration of 3D-ACAE in deep clustering

After DR, the image is fed into the 3D-ACAE model for feature extraction and deep clustering. The 3D-ACAE model comprises two main components: the encoder and the decoder. First, the HSI is divided into fixed-size cubes and subjected to DR. These cubes are then input to the encoder for spatial-spectral feature extraction through two 3D convolutional layers. Between these layers, specific regions of the HSI data are randomly deactivated using 3D spatial dropout to mitigate overfitting. Subsequent refinement is accomplished through the spatial-spectral attention module. The encoder’s output is flattened into a one-dimensional vector and connected to an embedding layer, which is another fully connected layer. The dimension of this layer is set to the number of data categories to encourage the encoder to extract features more conducive to clustering. The output of the embedding layer is re-scaled to its pre-compression dimension in the decoder path and reconstructed into a 3D spatial-spectral feature. The 3D transposed convolution is then applied to reconstruct the data and the 3D spatial dropout is also applied to mitigate overfitting.

In this section, there are two issues that need to be discussed in particular, the first is the loss function of deep clustering and the second is the spatial-spectral attention module.

#### Loss function of deep clustering

The mean square error (MSE) is computed between the reconstructed data and the input data, serving as the loss function for the network. The MSE is defined as follows:

9 $$\begin{aligned} L_{r}=\frac{1}{n} \sum _{i=1}^{n}\left\| g\left( f\left( x_{i}\right) \right) -x_{i}\right\| _{2}^{2} \end{aligned}$$

where *n* is the number of data in the dataset, $x_{i} \in \mathbb {R}^{3}$ is the *i*-th data in the dataset.

The output features from the embedding layer are also fed into the clustering layer, where the data is partitioned. We use the K-means++ algorithm to initialize the parameters in the clustering layer. Each data point $z_{i}$ is mapped to the soft label $q_{i}$ using Student’s *t-*distribution, which is defined as:

10 $$\begin{aligned} q_{i j}=\frac{\left( 1+\left\| z_{i}-\mu _{j}\right\| ^{2}\right) ^{-1}}{\sum _{j}\left( 1+\left\| z_{i}-\mu _{j}\right\| ^{2}\right) ^{-1}} \end{aligned}$$

where $q_{ij}$ is the *j*-th entry of $q_{i}$ and represents the probability that $z_{i}$ belongs to cluster *j*.

The clustering layer utilizes the KL divergence as the loss function (11), which optimizes the clustering process:

11 $$\begin{aligned} L_{c}=K L(P \Vert Q)=\sum _{i} \sum _{j} p_{i j} \log \frac{p_{i j}}{q_{i j}} \end{aligned}$$

where *P* is the target distribution, defined as:

12 $$\begin{aligned} p_{i j}=\frac{q_{i j}^{2} / \sum _{i} q_{i j}}{\sum _{j}\left( q_{i j}^{2} / \sum _{i} q_{i j}\right) } \end{aligned}$$

Therefore, the optimization objective of the proposed method is expressed as the combination of reconstruction and clustering losses:

13 $$\begin{aligned} L = L_{r} + \gamma L_{c} \end{aligned}$$

where $\gamma$ controls the degree of distortion of the embedded space.

This study employs stochastic gradient descent and backpropagation algorithms to update the parameters of the Convolutional Autoencoder (CAE) and the clustering centers. The target distribution *P* represents the actual ground truth soft labels, which are contingent upon the predicted soft labels. After every T iterations of Eqs. (9) and (11), an update is made to *P*.

**Figure 2:**
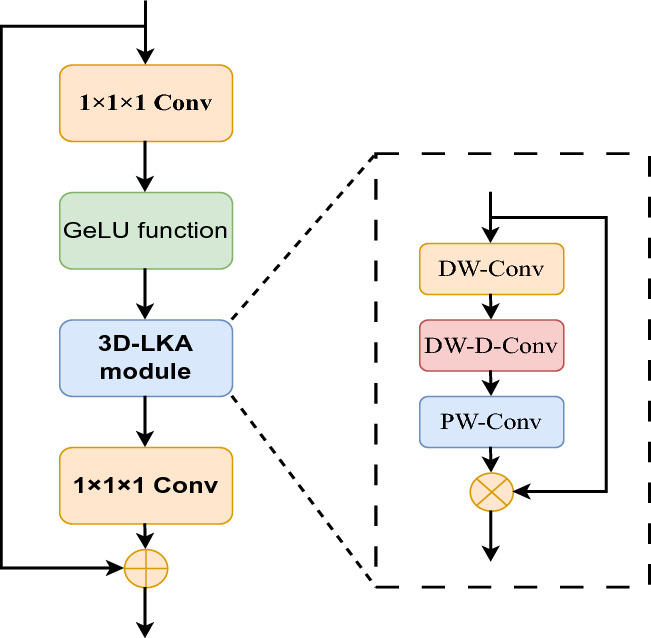
The structure of spatial–spectral attention module.

#### Spatial-spectral attention module

The attention mechanism enhances feature extraction, creating a detailed attention map. By combining this map with the feature map, it strengthens specific feature details, boosting discriminative qualities and overall model performance.

We enhance the accuracy and validity of the extracted features by integrating an attention module into our 3D-ACAE model (Fig. 2). Initially, the features undergo compression through a convolutional layer with a kernel size of $1 \times 1 \times 1$ for the number of channels, followed by the application of a Gaussian error linear unit (GeLU) non-linear activation function. Subsequently, the processed features are fed into the 3D large kernel attention (3D-LKA) module, where an attention mechanism is applied. The resulting feature map is then passed through another convolutional layer with a kernel size of $1 \times 1 \times 1$, restoring the original number of channels, and a residual connection is added to refine the features.

The 3D-LKA module comprises three components: local convolution (depth-wise convolution, DW-Conv), long-range convolution (depth-wise dilation convolution, DW-D-Conv), and channel convolution (point-wise convolution, PW-Conv). In our study, we implement long-range convolution using a dilation convolution to obtain long-range relationships within the data without introducing additional parameters. Channel adaptation is also essential at this stage because different channels in DNNs generally represent distinct objects. Therefore, we utilize PW-Conv for channel adaptation within the 3D-LKA model. The 3D-LKA module is expressed as:

14 $$\begin{aligned} Attention= & {} \text {PW-Conv}(\text {DW-D-Conv}(\text {DW-conv}(F))) \end{aligned}$$

15 $$\begin{aligned} Output= & {} Attention \otimes F \end{aligned}$$

where *F*, *Attention*, and $\otimes$ denote the input feature, attention map, and element -wise product, respectively.

In the experiments, the parameters of the LKA are configured as follows: the kernel size of the DW-conv is 5, the DW-D-conv is 1 and the dilation rate is 3. The PW-conv is 1. In addition, the convolutional layer in the model is set up to have 32 channels with a kernel size of (5, 3, 3).

## Experimental results and analysis

Here we evaluate the performance of our proposed method on three publicly available HSI datasets. The experiments are conducted by using Python 3.6 and Tensorflow 2.6 with the Keras 2.6 API on an AMD Ryzen 7 5800H CPU and NVIDIA Geforce RTX 3070 laptop GPU. We use the following three evaluation metrics to assess the performance of the model: overall accuracy (OA), normalized mutual information (NMI), and adjusted rand index (ARI)^36,40,41^.

To evaluate the effectiveness of the proposed method, we compare the 3D-ACAE deep clustering approach with two traditional clustering methods and three newly developed methods. The traditional clustering methods are fuzzy c-means (FCM)^42^ and spectral clustering (SC)^43^; the newly developed methods are graph convolutional optimal transport (GCOT)^40^, graph convolutional subspace clustering (GCSC)^41^ and recurrent neural network based deep clustering(DRNNC)^37^. GCOT leverages optimal transport (OT) to learn discrete transport coupling and create natural affinity matrices for spectral clustering. It utilizes OT probability to detect the graph’s edge, representing the original feature’s non-linear structure. GCSC transforms the self-expressive features of data into the non-Euclidean domain, producing a more robust graph embedding dictionary. DRNNC utilizes RNN to build autoencoder to apply deep clustering method. We ensure unbiased comparison by using the same sized data for all experiments, with a uniform patch size of 5.

### Description of datasets

The Indian Pines (IP) dataset was collected in 1992 using the AVIRIS sensor. It consists of $145\,\times \,145$ pixels with a spatial resolution of 20 m. The dataset contains 224 bands in the wavelength range of $0.4{-}2.5 \times 10^{-6}$ m, categorized into 16 groups. The number of bands was reduced to 200 by excluding those that covered the absorption area.

The Pavia University (PU) dataset was acquired in 2001 using the ROSIS sensor during a flight over the northern Italian region of Pavia University. The dataset comprises 103 bands in the range of $0.43{-}0.86 \times 10^{-6}$ m, with a spatial resolution of 1.3 m per pixel and it contains $610\times 340$ pixels. There are nine categories of land cover in the scene for classification.

**Figure 3:**
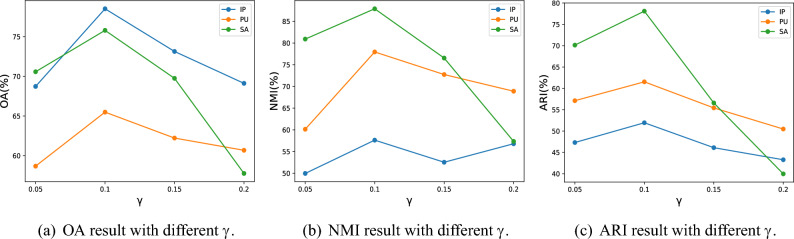
OA, NMI, and ARI results with different $\gamma$ values.

**Figure 4:**
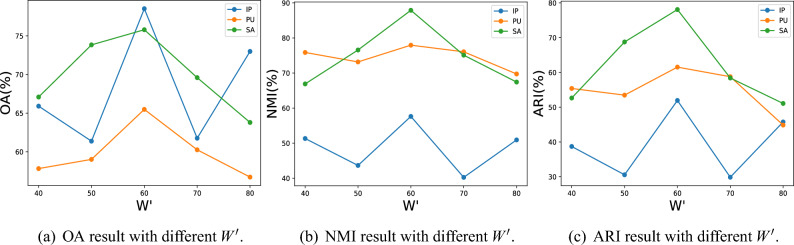
OA, NMI, and ARI results with different $W^{\prime }$ values.

Salinas valleyValley (SA) dataset, like the IP dataset, was acquired using the AVIRIS sensor over the Salinas Valley in California. It contains $512 \times 217$ pixels with a higher spatial resolution of 3.7 m per pixel. We eliminated some absorption bands from the dataset, resulting in 204 bands in the final dataset. The dataset contains 16 classes for land cover classification.

We followed these work^40,41,44–46^ and selected sub-scenes for the three datasets to ensure fairness in the comparison experiments. The sub-scenes of the IP, PU, and SA datasets used in this study are [30–115, 24–94], [591–676,158–240] and [150–350,100–200], respectively.

### Hyperparameters discussion

We conducted a discussion on two hyperparameters within the proposed method, namely, the spatial distortion control coefficient $\gamma$ in (13) and the dimensionality reduction target $W^\prime$. For $\gamma$, we conducted experiments with four representative values, specifically 0.05, 0.1, 0.15, and 0.2. The experimental results for different $\gamma$ values are presented in Fig. 3. It can be observed that when $\gamma$ is set to 0.05, all the metrics across the three datasets exhibit relatively poor performance. This occurs because, when $\gamma$ is too small, the impact of the clustering loss on the overall loss of the network model is minimal, which is detrimental to the extraction of features suitable for clustering. As $\gamma$ is increased to 0.1, there is a noticeable improvement in all three metrics. However, with a further increase in $\gamma$, the metrics exhibit a declining trend. This is due to the fact that when $\gamma$ takes on larger values, the influence of the clustering loss on the model parameters becomes more significant, leading to a decrease in the accuracy of the features extracted by the model. Therefore, for subsequent experiments, we set $\gamma$ to 0.1.

For $W^\prime$, we considered values of 40, 50, 60, 70, and 80 for discussion. On all three datasets, these metrics changed significantly as $W^{\prime }$ increased from 40 to 80. The experimental results are depicted in the Fig. 4. It can be seen that all metrics on all three datasets achieve their maximum values at $W^{\prime }=60$. The evaluation metrics show a lower performance when $W^{\prime }<60$ or $W^{\prime }>60$ than in the case of $W^{\prime }=60$. On both the PU dataset and the SA dataset, all three metrics show an increasing and then decreasing trend. This indicates that if the value of $W^{\prime }$ is obtained low, it will not provide enough spectral features for feature extraction, while if $W^{\prime }$ is obtained too high, some redundant bands in the data will hurt the extracted features. Therefore, we choose $W^{\prime }$ as 60.

**Table 1:** Ablation experimental results of the HSI dataset clustering.

Methods	IP			PU			SA		
	OA	NMI	ARI	OA	NMI	ARI	OA	NMI	ARI
3D-CAE	65.86	41.89	32.32	58.76	63.08	43.22	53.80	58.35	35.02
3D-CAE with PCA	69.23	48.56	36.96	58.01	66.98	55.95	62.92	60.74	43.10
3D-ACAE	76.02	57.57	48.94	59.95	72.50	54.86	74.85	80.30	70.81
3D-ACAE with PCA	76.95	57.10	50.50	63.75	75.15	46.98	75.00	82.65	66.51
Proposed method	**78.52**	**57.63**	**51.95**	**65.49**	**77.95**	**61.54**	**75.80**	**87.89**	**78.10**

Best result values are indicated in bold.

### Ablation experiment

We conducted ablation experiments on three datasets to validate the effectiveness of the proposed method. The experiments were divided into five groups: 3D-CAE without any modification, 3D-CAE after data preprocessing using PCA, 3D-ACAE with only the addition of the attention mechanism, 3D-ACAE with preprocessing using PCA, and 3D-ACAE with the addition of the attention mechanism and DR using PCA and t-SNE. The results of these ablation experiments are presented in the Table 1. Regarding dimensionality reduction, the proposed method, as compared to 3D-ACAE employing PCA for dimensionality reduction, exhibited improvements of 1.57%, 1.74%, and 0.8%, respectively. This underscores that our PCA+t-SNE approach incurs less information loss when reducing the dimensionality of HSI compared to traditional methods, making it more conducive for subsequent processing.

**Figure 5:**
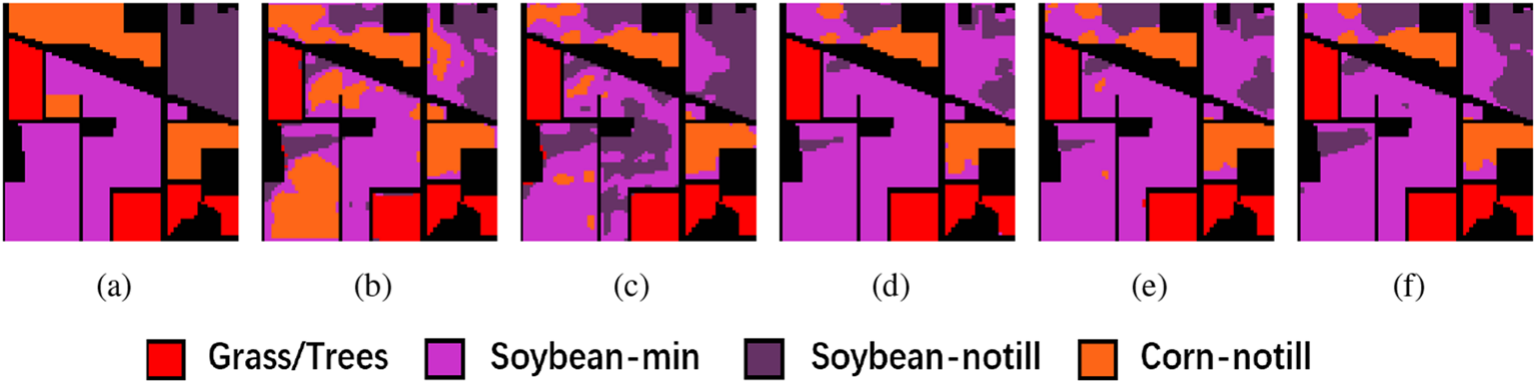
Ablation experimental results of the IP dataset. (**a**) Ground truth, (**b**) 3D-CAE, (**c**) 3D-CAE with PCA, (**d**) 3D-ACAE, (**e**) 3D-ACAE with PCA, and (**f**) the proposed method.

Concerning the incorporation of the spatial-spectral attention module into the model, the proposed 3D-ACAE, compared to the unmodified 3D-CAE, demonstrated improvements in OA of 10.16%, 1.19%, and 21.05%, respectively, across the three datasets. The NMI also exhibited enhancements of 15.68%, 9.42%, and 21.95%, and the ARI increased by 16.62%, 11.64%, and 35.79%, respectively. These findings confirm the significant effectiveness of the spatial-spectral attention module in refining the features extracted by the 3D-CAE, resulting in a substantial enhancement of deep clustering performance for HSI.

**Figure 6:**
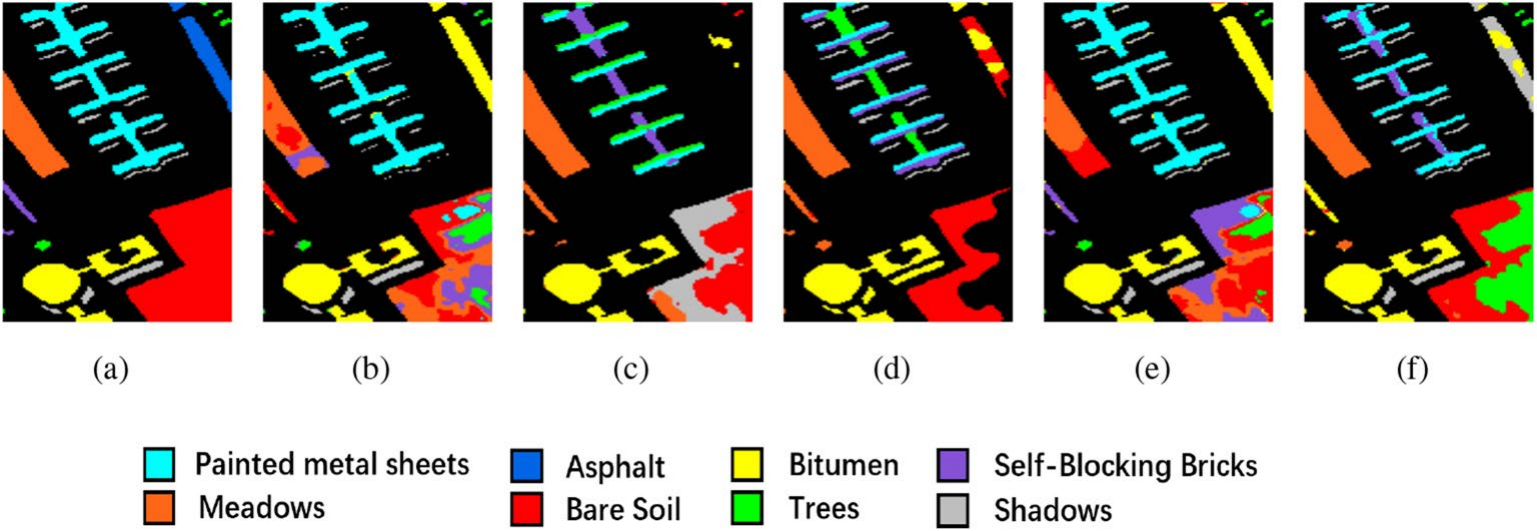
Ablation experimental results of the PU dataset. (**a**) Ground truth, (**b**) 3D-CAE, (**c**) 3D-CAE with PCA, (**d**) 3D-ACAE, (**e**) 3D-ACAE with PCA, and (**f**) the proposed method.

**Figure 7:**
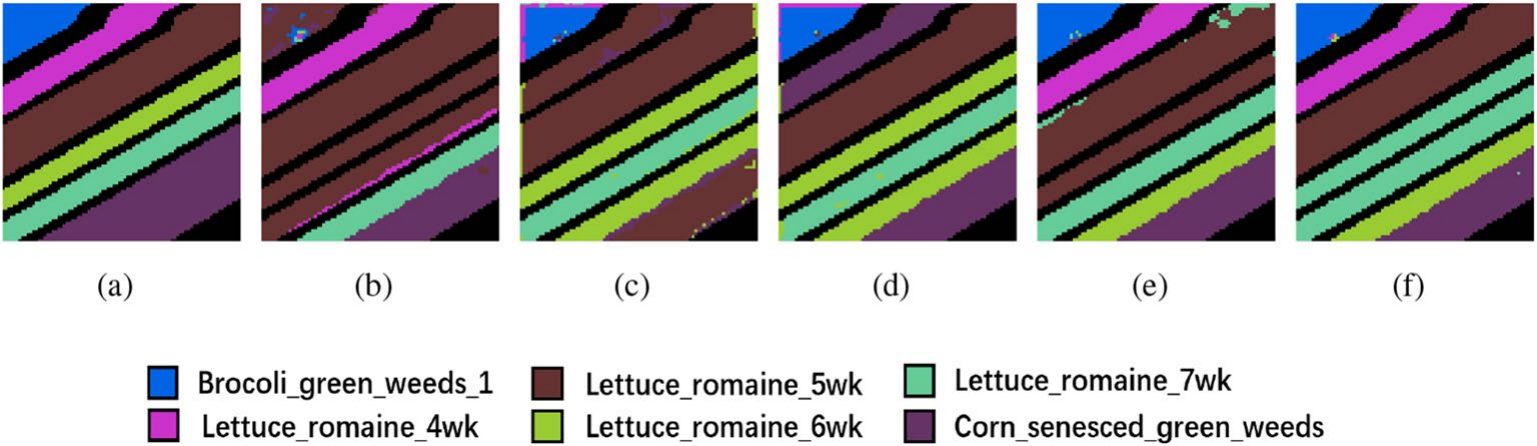
Ablation experimental results of the SA dataset. (**a**) Ground truth, (**b**) 3D-CAE, (**c**) 3D-CAE with PCA, (**d**) 3D-ACAE, (**e**) 3D-ACAE with PCA, and (**f**) the proposed method.

The clustering maps generated by the proposed method further illustrate its superiority. Across the three datasets, these maps produced by the proposed method are notably smoother when compared to the unimproved methods. For instance, in Fig. 5, the Soybean-min region is markedly smoother in the proposed method, while other clustering maps exhibit roughness within their regions, with the proposed method aligning closely with the ground truth. Similarly, in Fig. 6, the Bare soil region in the proposed method contains only two distinct colors with clear boundaries, in contrast to the other clustering maps, which include three or more colors and exhibit greater spatial disorder. And in Fig. 7, the proposed method is the most similar to ground truth, and the various other methods suffer from some degree of distortion. These observations collectively substantiate the effectiveness of the proposed method in the task of HSI clustering.

**Table 2:** Clustering results of IP dataset.

	FCM	SC	GCSC	GCOT	DRNNC	Proposed
Soybean-min	54.48	76.67	60.79	36.7	56.74	**79.94**
Grass/trees	98.35	80.6	99.04	98.64	97.73	**99.45**
corn-notill	60.64	56.32	59.84	60.4	0	**61.34**
Soybean-notill	13.45	12.09	19.87	**75.69**	39.48	58.76
OA	54.82	68.12	59.74	60.31	49.92	**78.52**
NMI	39.28	53.15	47.58	45.43	35.28	**57.63**
ARI	32.76	37.56	34.50	32.48	24.45	**51.95**

Best result values are indicated in bold.

**Table 3:** Clustering results of PU dataset.

	FCM	SC	GCSC	GCOT	DRNNC	Proposed
Painted metal sheets	35.46	35.32	38.46	**99.69**	87.66	61.48
Asphalt	0	0	0	0	0	**0**
Bitumen	78	97.93	97.85	96.33	95.42	**98.67**
Self-blocking bricks	24.22	0	0	11.32	**37.48**	14.46
Meadows	96.77	100	43.43	53.48	98.98	**100**
Bare soil	30.65	26.78	47.78	24.77	24.68	**54.68**
Trees	0	**99.32**	0	89.96	24.36	67.66
Shadows	97.4	96.99	97.09	96.73	96.48	**97.64**
OA	54.37	53.31	62.05	62.02	54.960	**65.49**
NMI	66.86	73.10	70.24	75.70	61.55	**77.95**
ARI	46.18	48.01	49.68	55.14	41.24	**61.54**

Best result values are indicated in bold.

**Table 4:** Clustering results of SA dataset.

	FCM	SC	GCSC	GCOT	DRNNC	Proposed
Brocoli_green_weeds 1	0	0	97.43	96.54	97.87	**98.75**
Lettuce_romaine_4wk	74.36	96.74	**99.96**	0	78.64	97.7
Lettuce_romaine_5wk	99.4	98.78	99.53	100	97.77	**100**
Lettuce_romaine_6wk	**94.35**	100	78.59	0	85.43	21.45
Lettuce_romaine_7wk	**98.76**	95.69	43.43	0	20.58	87.9
Corn_seneed_green_weeds	68.48	75.8	25.46	100	21.46	**80.19**
OA	75.08	67.03	72.87	60.10	64.36	**75.80**
NMI	80.59	79.07	75.21	63.79	70.10	**87.89**
ARI	74.76	57.53	65.60	39.26	60.34	**78.10**

Best result values are indicated in bold.

### Comparison experimental results

The clustering results (Tables 2, 3, 4; best outcomes highlighted in bold) of comparison methods on various datasets indicate that the proposed method achieves the highest performance. This finding verifies the viability of integrating deep learning with conventional clustering algorithms in the HSI deep clustering domain.

The Tables 2, 3, 4 clearly indicates that the proposed method has achieved the best results across all datasets in terms of its three evaluation metrics. For the IP dataset, the OA stands at 78.52%, representing a notable improvement of 28.6% over the worst-performing method, DRNNC, and a 10.4% improvement over the best-performing method, SC. Furthermore, the proposed method outperforms these two methods in terms of NMI, with improvements of 22.35% and 4.48%, respectively. Turning to the PU dataset, the proposed method showcases an OA of 65.49%, along with NMI and ARI values of 77.95% and 61.54%, respectively. These results signify improvements of 3.44% (GCSC), 2.25% (GCOT), and 6.4% (GCOT) over the methods that perform best on these three metrics. In the case of the SA dataset, the proposed method attains an OA of 75.80%, NMI of 87.89%, and ARI of 78.10%, indicating enhancements of 0.72%, 7.3%, and 3.34%, respectively, over the top-performing method, FCM, across these three metrics.

Tables 2, 3 and 4 also report the results of the accuracy of each category (CA) in the three datasets. According to CA, our proposed method gives better results for most of the categories, and a small number of categories are misclassified because of the great similarity of spatial-spectral features between categories, such as Soybean-notil and Soybean-min in IP, and Asphalt and Bitumen in PU. Overall, the results of CA also prove the effectiveness of the proposed method.

**Figure 8:**
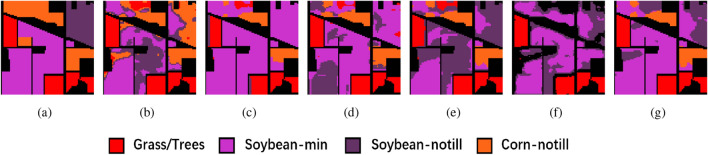
Clustering maps of the IP dataset. (**a**) Ground truth, (**b**) FCM, (**c**) SC, (**d**) GCSC, (**e**) GCOT, (**f**) DRNNC, and (**g**) the proposed method.

**Figure 9:**
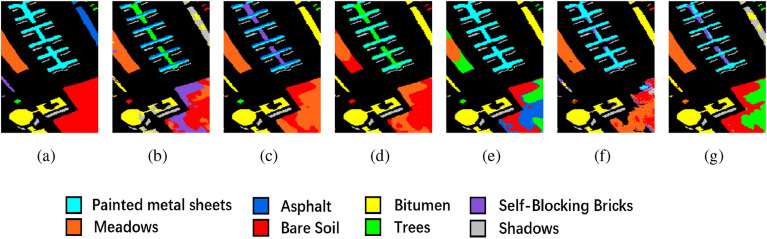
Clustering maps of the PU dataset. (**a**) Ground truth, (**b**) FCM, (**c**) SC, (**d**) GCSC, (**e**) GCOT, (**f**) DRNNC, and (**g**) the proposed method.

**Figure 10:**
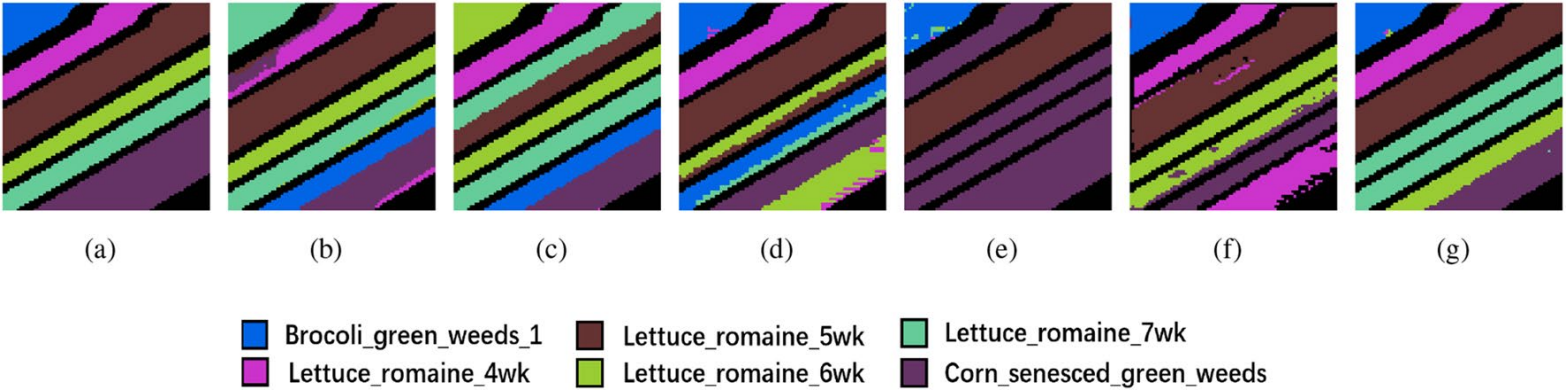
Clustering maps of the SA dataset. (**a**) Ground truth, (**b**) FCM, (**c**) SC, (**d**) GCSC, (**e**) GCOT, (**f**) DRNNC, and (**g**) the proposed method.

The improved clustering accuracy achieved by the proposed method is also reflected in the clustering maps it generates. In the IP dataset, as depicted in Fig. 8, the Soybean-min region is notably more complete when compared to other methods, and the Corn-notill region also exhibits closer proximity to the ground truth. In the PU dataset, the Meadows region in Fig. 9 is the smoothest among all comparative methods. For the SA dataset, the clustering map generated by the proposed method, as shown in Fig. 10, closely aligns with the ground truth, further validating the substantial performance enhancement offered by the proposed method in clustering accuracy.

We also compared the running times of different methods, as shown in Table 5. Among all the methods, the two traditional approaches, FCM and SC, exhibited the fastest execution times. GCSC and GCOT, utilizing graph convolution for computation, required slightly more time than the two aforementioned algorithms. In contrast, both DRNNC and the proposed method, involving deep learning, necessitated model training and parameter adjustment through backpropagation. As a result, they incurred the longest execution times. Nevertheless, given the substantial improvement in clustering performance, the increase in execution time is acceptable.

**Table 5:** Comparison of running times of different clustering methods.

Dataset	FCM	SC	GCSC	GCOT	DRNNC	Proposed method
IP	0.358	2.762	16.382	3.708	106.888	258.104
PU	0.487	2.819	33.344	14.285	103.314	130.791
SA	0.423	1.873	25.709	6.759	103.511	256.604

## Conclusion

This study presents a novel deep clustering approach for HSI analysis. The proposed method is first applied PCA and t-SNE to reduce the dimensionality of the original HSI. Subsequently, a novel 3D-ACAE model is constructed to extract spatial-spectral features from the dimension-reducted HSI data. To refine the extracted features and improve the representation for the HSI, a spatial-spectral attention module is incorporated. The model integrates a clustering layer after the embedding layer to obtain clustering results. The parameters of the clustering layer are initialized using K-means and optimized through the backpropagation algorithm. Experimental results demonstrate the feasibility of deep clustering for HSI analysis. In future work, the deep clustering approach will be combined with superpixel segmentation to explore the spatial and spectral information in HSI more comprehensively.

## Data Availability

The datasets analysed during the current study are available in the Search Computational Intelligence Group repository, https://www.ehu.eus/ccwintco/index.php?title=Hyperspectral_Remote_Sensing_Scenes.
